# Biomarkers of Intestinal Injury and Dysfunction: Adding New Possibilities to Current Methods for Risk Stratification of Children with Malaria Disease

**DOI:** 10.1128/mbio.02222-22

**Published:** 2022-10-31

**Authors:** Núria Balanza, Elisa López-Varela, Bàrbara Baro, Quique Bassat

**Affiliations:** a ISGlobal, Hospital Clínic, Universitat de Barcelona, Barcelona, Spain; b Centro de Investigação em Saúde de Manhiça, Maputo, Mozambique; c ICREA, Barcelona, Spain; d Pediatrics Department, Hospital Sant Joan de Déu (University of Barcelona), Barcelona, Spain; e Consorcio de Investigación Biomédica en Red de Epidemiología y Salud Pública (CIBERESP), Madrid, Spain

**Keywords:** malaria, intestinal injury, gut barrier dysfunction, pediatrics, mortality

## Abstract

Malaria remains, in 2022, a major cause of pediatric preventable mortality, with its major burden disproportionately circumscribed to sub-Saharan African countries. Although only ~1 to 2% of malaria cases can be considered severe and potentially life threatening, it is often challenging to identify them so as to prioritize adequate health care and resources. In a recent investigation, M. L. Sarangam, R. Namazzi, D. Datta, C. Bond, et al. (mBio 13:e01325-22, 2022, https://journals.asm.org/doi/10.1128/mbio.01325-22) studied intestinal barrier dysfunction and injury in Ugandan children hospitalized with severe malaria and in healthy community controls. By measuring circulating levels of four different and complementary biomarkers of gut barrier dysfunction and microbial translocation, they demonstrated that intestinal injury is common in pediatric severe malaria (18% of all cases) and is associated with increased mortality, acute kidney injury, acidosis, and endothelial activation. This commentary discusses the prognostic implications of these results, knowledge gaps that remain to be filled, and how findings could be potentially translated into effective interventions to improve outcomes in children with malaria.

## COMMENTARY

Malaria remains a major global health problem, and the COVID-19 pandemic has further aggravated a situation which, since 2015, has certainly been at a crossroads (1). Of the circa 241 million cases of malaria that were estimated to occur in 2020, a minimum of 2.5 million were severe ones, which led to an estimated 627,000 deaths worldwide. Children under 5 years of age are the group at most risk, disproportionately accounting for up to 77% of all deaths (2). In a delicate equilibrium between the exposure to *Plasmodium* parasites (determined by intensity of transmission), host and parasite factors, and the natural progressive acquisition of partial immunity, malaria shows a wide spectrum of clinical presentations, ranging from purely asymptomatic infections to rapidly evolving life-threatening disease. Severe malaria (SM) is a multisystem disease, which manifests as several overlapping syndromes such as impaired consciousness, respiratory distress, severe anemia, and acute kidney injury (AKI). Nevertheless, our current understanding of SM pathogenesis remains far from complete. Additionally, it is often challenging to predict or identify which patients with malaria are at higher risk of developing severe disease, given the unspecific and overlapping nature of clinical symptoms, the scarcity of prognostic tools in the settings where malaria is endemic, and the potential for this disease to evolve rapidly. Thus, any rapid triaging tool that could help determine, objectively, quantitatively, and with high precision, which malaria patients are at high risk of dying so as to prioritize their care would be an invaluable addition to our limited arsenal to decrease malaria-associated mortality.

Gastrointestinal symptoms can be frequent in malaria (3, 4), yet there has been little interest in exploring intestinal injury and its role in SM pathogenesis. Histopathological studies of fatal cases have demonstrated intense sequestration of infected erythrocytes in the intestinal microvasculature and small bowel intussusceptions (5, 6). *In vivo* studies in malaria cases have shown increased gastrointestinal permeability (7) and metabolomic analysis suggested compromised intestinal barrier function in SM (8). Studies in mice have described transient gut dysbiosis after *Plasmodium* infection, as well as an association between distinct gut microbiomes and malaria severity (9). Actually, translocation of enteric bacteria is hypothesized to be a common explanation of concomitant invasive bacterial infection during malaria, which substantially worsens prognosis (10, 11). Could gut barrier dysfunction be a key but insufficiently recognized piece of the puzzle in SM pathogenesis?

The recent investigation by Sarangam and colleagues (12) explored intestinal barrier dysfunction in 598 Ugandan children hospitalized with SM and 120 healthy community controls, aged 6 months to 4 years. They measured in serum or plasma a biomarker of enterocyte damage (intestinal fatty acid binding protein [I-FABP]), a biomarker of intestinal mucosal maintenance and repair (trefoil factor 3 [TFF3]), and two biomarkers that may reflect microbial translocation from the gut lumen into the circulation (lipopolysaccharide binding protein [LBP] and soluble complement of differentiation 14 [sCD14]). In order to further explore biomarker kinetics, measurements were conducted upon admission and repeated after 1 month during the follow-up clinical visit. The authors showed that intestinal injury was a common feature at hospital admission in this SM pediatric cohort, with 18% of children fulfilling the definition of either elevated TFF3 or I-FABP compared to the population reference levels (i.e., >99th percentile for the healthy population). As expected, intestinal injury was associated with gastrointestinal symptoms. All four biomarker levels were significantly elevated in children with SM upon admission compared to healthy community children. All, except TFF3, decreased after 1 month to reach levels comparable to those of community children. These findings support the notion that an important proportion of children with SM suffer from intestinal barrier dysfunction when admitted to the hospital, which rapidly improves in the weeks after, leaving only evidence of mucosal healing.

Children with intestinal injury at admission were more likely to have clinical signs consistent with increased disease severity, AKI, acidosis, and elevated biomarkers reflecting endothelial activation. Importantly, TFF3 and I-FABP levels at admission were associated with in-hospital mortality, and TFF3 levels also were associated with 12-month postdischarge mortality. In pediatric SM, as in other infectious diseases, few biomarkers indicative of endothelial and immune activation have been consistently reported to be associated with mortality (13–15). Biomarkers hold potential as risk stratification tools, as they can be measured by standard laboratory methods or be incorporated into easier-to-use point-of-care devices and inform about risk of adverse outcomes in an objective and quantitative manner. However, whether TFF3 and I-FABP have better prognostic accuracy than other promising prognostic markers remains unknown. Unfortunately, the authors did not use their extensive biomarker data to answer this question in their report. Future research should include comparative studies and evaluate if TFF3 and I-FABP could effectively be used to improve risk stratification of children with SM and determine whether other less invasive samples beyond peripheral blood (for instance, stool) could be used to ascertain biomarker levels and subsequent risk.

Intestinal injury in SM is hypothesized to be caused by parasite sequestration in intestinal capillaries, hypoperfusion secondary to different pathophysiological processes, malaria-induced systemic immune and endothelial activation, underlying factors related to the host microbiome, or a combination of these and other complex mechanisms. A significant highlight of the work of Sarangam et al. is undoubtedly the description of the interrelation of intestinal injury, AKI, and acidosis, in an attempt to further understand the role of intestinal injury in SM. Interestingly, they reported that intestinal injury overlapped significantly with AKI and acidosis, and those children presenting with all three features had an increased risk of in-hospital mortality. Different shared pathways could explain this three-way interaction, as discussed in detail by the authors. Of note, markers of intestinal injury and markers of microbial translocation (including blood culture positivity) were surprisingly not correlated in this cohort. Although further studies are warranted to disentangle the underlying pathological mechanisms, these findings are already key and importantly advance our current knowledge on pediatric SM pathogenesis.

Remarkably, Ngai and colleagues (16) recently published a similar study that complements, validates, and further expands the findings of Sarangam et al. The biomarkers I-FABP, LBP, and sCD14 (in addition to zonula occludens-1 [ZO-1], another marker of intestinal injury) were studied in a different cohort of 523 Ugandan children hospitalized with SM. That study showed that severe intestinal injury, defined slightly differently (i.e., I-FABP levels of ≥5.6 ng/mL), was present in 1 out of every 10 sick children. I-FABP was also associated with gastrointestinal symptoms, impaired tissue perfusion, AKI, central nervous system dysfunction, inflammation, and endothelial activation. Moreover, I-FABP was associated with in-hospital death as well. Given the high prevalence of intestinal injury and its association with mortality demonstrated by both studies, findings hint toward the need to not only explore the prognostic accuracy of intestinal injury markers but also use the current unveiling mechanistic and pathophysiological understanding to conduct clinical trials of agents that stabilize the intestinal barrier. Mitigating intestinal damage, as an adjunctive therapy strategy in pediatric SM, could improve recovery and survival in the most vulnerable children.

Nevertheless, multiple questions remain unanswered and should be addressed by future analogous biomarker studies. Does this phenomenon of intestinal injury happen in populations of older children or adults with SM? Is it already present to some extent in uncomplicated malaria cases? Is this also occurring in other life-threatening infections, and therefore, could this be a “pathogen-agnostic” mechanism of severe disease? Even if there is much work ahead, one thing is clear: we are now one step closer to understanding the mysteries of SM and to improving outcomes in malaria patients by turning these new insights into innovative and successful interventions.
